# Near infrared light induced plasmonic hot hole transfer at a nano-heterointerface

**DOI:** 10.1038/s41467-018-04630-w

**Published:** 2018-06-13

**Authors:** Zichao Lian, Masanori Sakamoto, Hironori Matsunaga, Junie Jhon M. Vequizo, Akira Yamakata, Mitsutaka Haruta, Hiroki Kurata, Wataru Ota, Tohru Sato, Toshiharu Teranishi

**Affiliations:** 1 Department of Chemistry, Graduate School of Science, Kyoto University, Gokasho, Uji, Kyoto 611-0011 Japan; 2 Institute for Chemical Research, Kyoto University, Gokasho, Uji, Kyoto 611-0011 Japan; 3 Graduate School of Engineering, Toyota Technological Institute, 2-12-1 Hisakata, Tempaku, Nagoya 468-8511 Japan; 4 Department of Molecular Engineering, Graduate School of Engineering, Kyoto University, Nishikyo-ku, Kyoto 615-8510 Japan; 5 Fukui Institute for Fundamental Chemistry, Kyoto University, Takano-Nishihiraki-cho 34-4, Sakyo-ku, Kyoto 606-8103 Japan; 6 Unit of Elements Strategy Initiative for Catalysts and Batteries, Kyoto University, Nishikyo-ku, Kyoto 615-8510 Japan

## Abstract

Localized surface plasmon resonance (LSPR)-induced hot-carrier transfer is a key mechanism for achieving artificial photosynthesis using the whole solar spectrum, even including the infrared (IR) region. In contrast to the explosive development of photocatalysts based on the plasmon-induced hot electron transfer, the hole transfer system is still quite immature regardless of its importance, because the mechanism of plasmon-induced hole transfer has remained unclear. Herein, we elucidate LSPR-induced hot hole transfer in CdS/CuS heterostructured nanocrystals (HNCs) using time-resolved IR (TR-IR) spectroscopy. TR-IR spectroscopy enables the direct observation of carrier in a LSPR-excited CdS/CuS HNC. The spectroscopic results provide insight into the novel hole transfer mechanism, named plasmon-induced transit carrier transfer (PITCT), with high quantum yields (19%) and long-lived charge separations (9.2 μs). As an ultrafast charge recombination is a major drawback of all plasmonic energy conversion systems, we anticipate that PITCT will break the limit of conventional plasmon-induced energy conversion.

## Abstract (web summary)

Hot hole transfer has applications in plasmonics, photocatalysis, and light harvesting, but is often limited by low quantum yields and short-lived charge separation times. Here, Lian et al. overcome these limitations in heterostructured nanocrystals and proposed a new hot hole transfer mechanism.

## Introduction

Localized surface plasmon resonance (LSPR)-induced photoenergy conversion is among the great challenges causing a paradigm shift in both scientific fields and industry regarding solar-energy utilization^1–5^. The LSPR band can be tuned over a wide spectral range by changing the carrier density, morphology, and other material properties, enabling solar energy utilization from ultraviolet (UV) to infrared (IR) regions. Although plasmonic materials have superior light-harvesting abilities, the low conversion efficiency caused by ultrafast relaxation of the hot carrier and charge recombination is a major drawback. Hot carrier transfer competes with ultrafast relaxation via carrier scattering with timescales of just hundreds of femtoseconds (fs) ^6,7^, and recombination after the charge separation completes in picoseconds (ps) region. Therefore, it is difficult to achieve sufficient extraction of hot carriers for practical applications from the kinetic perspective^8,9^. Furthermore, the unclear behavior of hot holes, which has remained an obscure subject regardless of the unmistakable importance, becomes an obstacle for the comprehensive understanding of LSPR-induced carrier transfer.

Recently, a novel series of compound semiconductors, copper chalcogenide nanocrystals, which show excellent tunable hole-based LSPR absorption in the near-IR (NIR) region, have attracted much attention as candidates for IR-responsive photocatalysts^10,11^. For heterostructured nanocrystals (HNCs) composed of plasmonic copper sulfide (CuS) phase and another metal or semiconductor phase (for example, acceptor phase), hot hole transfer has been proposed as a possible mechanism for providing IR-induced catalytic activity. However, the mechanism has yet to be determined. Elucidation of this mechanism would clarify the role of hot holes contributing the photocatalytic activity^12^.

Herein, we elucidate the LSPR-induced behavior of hot holes in CuS NCs and CdS/CuS HNCs using time-resolved infrared (TR-IR) spectroscopy. TR-IR spectroscopy enables us the direct observation of carrier in a photo-excited nanocrystal. The CdS/CuS HNCs are one of the promising combinations for the spectroscopic tracing of the LSPR-induced hole transfer from the CuS phase to the CdS phase. We discover in the detailed investigation that a multi-step carrier transfer (plasmon-induced transit carrier transfer: PITCT) realized efficient hole transfer from the CuS phase to the CdS phase. Surprisingly, the PITCT of CdS/CuS HNCs achieves high quantum yields (19%) and long-lived charge separations (9.2 μs), which has not been observed in plasmon-induced carrier-injection systems. Because ultrafast charge recombination is a major drawback of all plasmonic energy conversion systems, the PITCT mechanism proposes here should change the conventional consensus regarding LSPR-induced energy conversion due to the overwhelming advantage of high hot carrier transfer efficiency caused by in situ trapping of hot carriers and long-lived charge separation.

## Results

### Characterization of materials

**Fig. 1:**
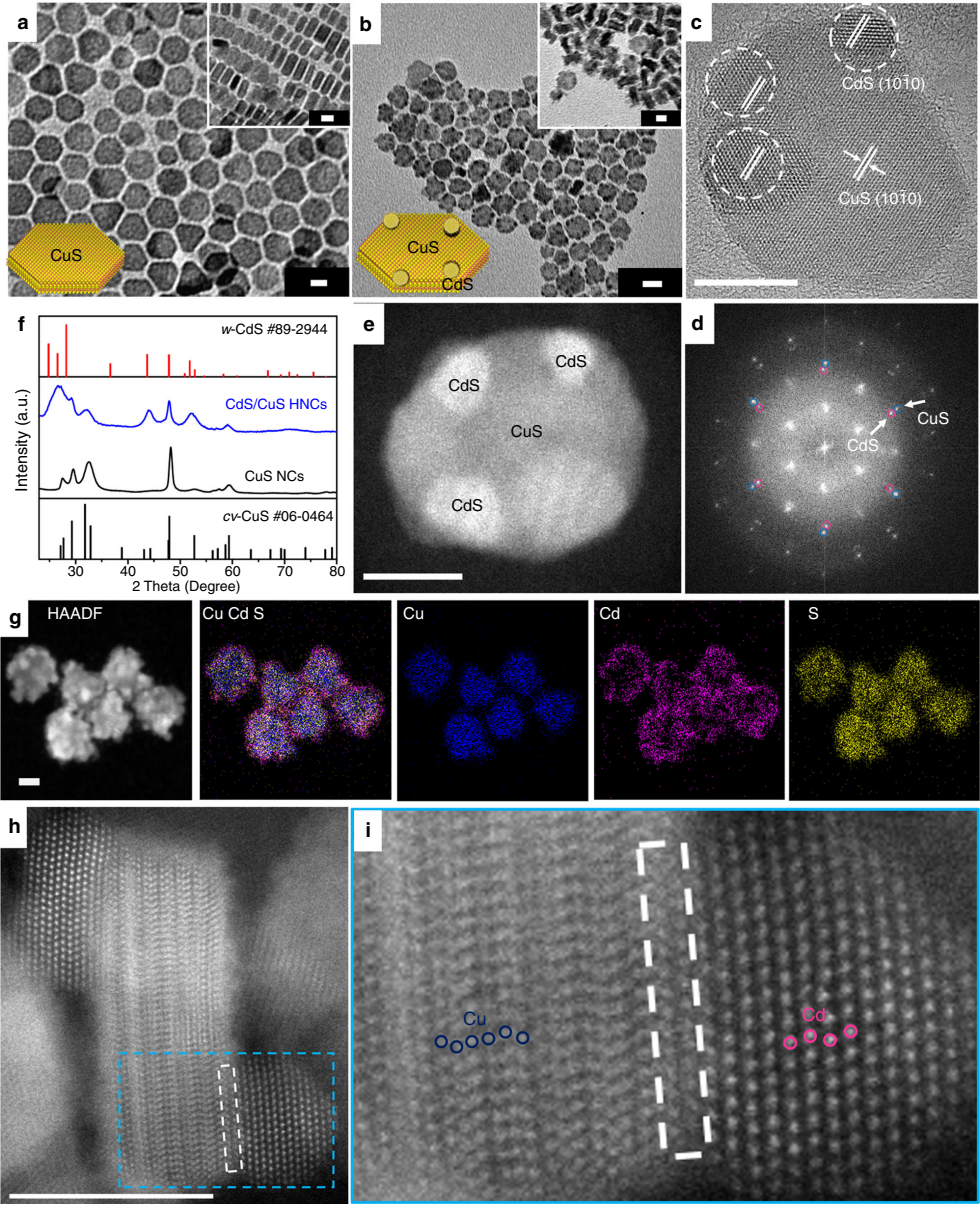
Structural characterization of the nanocrystals. **a**, **b** Representative transmission electron microscopy (TEM) images of **a** plate-shaped CuS nanocrystals (NCs) (inset: stacked CuS NCs), **b** CdS/CuS herostructured NCs (HNCs) (inset: stacked CdS/CuS HNCs). **c** High-resolution TEM (HRTEM) image of CdS/CuS HNCs from the *c* axis. **d** Fast Fourier transform (FFT) patterns of a single CdS/CuS HNC from **c**. **e** High-angle annular dark-field scanning TEM (HAADF-STEM) image of a single CdS/CuS HNC. **f** X-ray diffraction (XRD) patterns of CuS NCs and CdS/CuS HNCs. **g** HAADF-STEM energy dispersive X-ray spectrometry (EDS) elemental mapping images of CdS/CuS HNCs. **h** HAADF-STEM image of stacked CdS/CuS HNCs, where the interface is labeled with a dashed white rectangle and the electron beam incident direction is parallel to [11–20]. **i** Enlarged part of **h** with a dashed blue rectangle, showing Cu and Cd atomic arrangements. Scale bars: 10 nm

We synthesized CdS/CuS HNCs using plate-shaped CuS NCs as seeds (see details in the Supplementary Figs. 1–5 and Supplementary Note 1). Figure 1a shows transmission electron microscopy (TEM) images of monodisperse CuS NCs (size, 16.3 ± 1.5 nm; thickness, 5.7 ± 1.1 nm). When CdS phases were grown on the CuS NCs, multiple CdS satellites of 3.8 ± 0.8 nm in size were deposited on the peripheral regions of the CuS NCs, as shown in Fig. 1b. The X-ray diffraction (XRD) patterns in Fig. 1f clearly show that the CdS/CuS HNCs were composed of hexagonal covellite CuS (*cv*-CuS, Joint Committee on Power Diffraction Standards (JCPDS) no. 06–0464) and wurtzite CdS (*w*-CdS, JCPDS no. 89–944) phases, with the Cd/Cu molar ratio determined as 38:62 by X-ray fluorescence (XRF) spectroscopy.

The high-resolution TEM (HRTEM) image in Fig. 1c shows the high crystallinity of the CdS/CuS HNCs and the different lattice fringes corresponding to *w*-CdS (10–10) and *cv*-CuS (10–10) lattices. The fast Fourier transform (FFT) of Fig. 1c shows splitting spots that correspond to the CdS and CuS phases (Fig. 1d) and are aligned toward the same radial direction from the central spot, indicating the epitaxial growth of CdS on the CuS phase. High-angle annular dark-field (HAADF) scanning TEM (STEM) (Fig. 1e) and STEM-energy dispersive X-ray spectrometry (EDS) mapping (Fig. 1g) also showed that the CuS NCs were surrounded by multiple CdS satellites.

To clarify the atomic arrangement at the interface between *w*-CdS and *cv*-CuS, HAADF-STEM measurements were carried out as shown in Fig. 1h, i^13^. In the HAADF-STEM image, since only Cd and Cu atomic columns in *w*-CdS and *cv*-CuS were clearly observed but S columns were not observed, it is impossible to distinguish the polarity. The CuS and CdS phases had orientation relationships of *w*-CdS [0001]//*cv*-CuS [0001] and *w*-CdS [11–20]//*cv*-CuS [11–20], which resulted in intimate contact between the two phases. It is considered that *cv*-CuS (0001) and (000-1) planes were terminated by S anion from the view point of the atomic arrangement of Cu ion. And it seems that S anions are connected to Cd^2+^-terminated *w*-CdS (0001) planes with a lattice mismatch of 8.33% and *w*-CdS phases are epitaxially grown on the *cv*-CuS NCs to form CdS/CuS HNCs.

### Optical properties of materials and TR-IR measurements

**Fig. 2:**
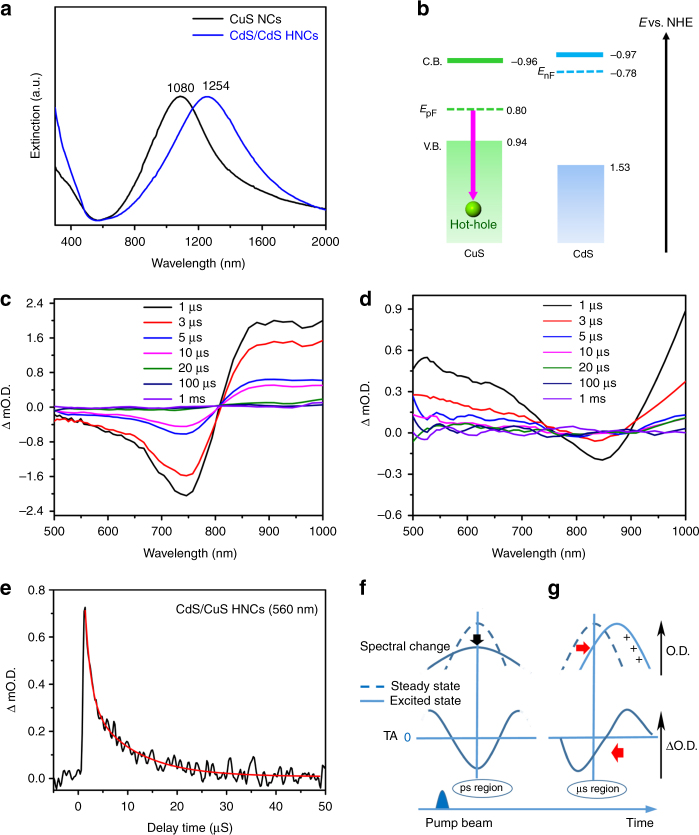
Extinction spectra, band diagrams, TR-IR spectra, and a schematic illustration of time-dependent changes in TA. **a** Extinction spectra of CuS nanocrystals (NCs) and CdS/CuS heterostructured NCs (HNCs) in hexane. **b** Band diagrams of CuS and CdS phases, *E*_pF_, *E*_nF_: Fermi level, the red arrow means plasmon excitation by near-infrared (NIR) light. **c**, **d** Time resolved IR spectroscopy (TR-IR) spectral changes for **c** CuS NCs and **d** CdS/CuS HNCs from visible to near-infrared regions in the microseconds (μs) region. **e** Kinetic profile of transient absorption (TA) for CdS/CuS HNCs at a specific probing wavelength (560 nm) tracking holes generated in the CdS phase by NIR local surface plasmon resonance (LSPR) excitation. Red line is the best fit. Pumping wavelength: 1200 nm. The ΔmO.D. means the change of optical density. **f**, **g** Schematic illustration of spectral changes at different timescales: **f** Bleaching of LSPR peak caused by hole and photon scattering in the CuS NCs observed in the ps region, and **g** surface hole trapping to reduce the number of holes in the intrinsic state, leading to a red-shift of the LSPR peak and blue shift of the bleaching position in the μs region

Next, we investigated LSPR-induced hole transfer from the CuS phase to the CdS phase. Figure 2a shows the extinction spectra of CuS NCs and CdS/CuS HNCs (see also Supplementary Fig. 1). The CuS NCs showed an LSPR peak at 1080 nm, while the LSPR peak of the CdS/CuS HNCs was red-shifted to 1254 nm. This red-shift of the LSPR peak might be attributed to the change of size, shape, and different dielectric environment with the existence of CdS phases^14^. Band diagrams of the CuS NCs and CdS NCs (Fig. 2b, Supplementary Figs. 6 and 7 for estimations of the conduction band (CB) and valence band (VB) positions) showed that the VB edge of the CdS NCs was 0.73 eV lower than the Fermi level of the CuS NCs (*E*_pF_), and could be accessed by hot holes generated by NIR LSPR in the CuS phases.

We conducted TR-IR measurements of CuS NCs and CdS/CuS HNCs in the μs region to investigate the LSPR-induced hole dynamics. Figure 2c and d show the TR-IR absorption spectra of the CuS NCs and CdS/CuS HNCs in the μs region after excitation of hole-based LSPR in the CuS phases with a 1200-nm laser (1.03 eV) to produce hot holes. The TR-IR spectra of the CuS NCs showed bleaching peaks at around 740 nm. In contrast, the TR-IR spectra of the CdS/CuS HNCs showed bleaching peaks at around 840 nm and absorption in the visible region. These transient absorption (TA) signals completely recovered within 1 ms after excitation, indicating that the observed spectral changes reflected a reversible process. As the LSPR-excitation-induced sequential event (that is, hole dephasing, hole–hole scattering, hole–phonon coupling, and lattice heat dissipation) was completed in the ps–ns region^3,15,16^, the observed spectral changes cannot be explained by the conventional LSPR-induced decay process. Furthermore, the bleaching peak was significantly blue-shifted compared with the LSPR peak. Therefore, we considered that this TR-IR spectral change originated from the reduction in the number of holes in the intrinsic state by trapping.

As the LSPR of compound semiconductors, which have a smaller number of carriers than metals, are sensitive to changes in carrier density, the redistribution of holes by trapping would cause a significant shift in the LSPR band^17–19^. Figures 2f and g show a schematic illustration of the spectral change and corresponding TA in different time regions. In the ps region, the aforementioned LSPR-excitation-induced sequential events caused LSPR bleaching (Fig. 2f)^11^. When some of the hot holes were trapped at trapping sites, the number of holes in the intrinsic state decreased, causing the red-shift in the LSPR peak, which was responsible for the significant blue-shift of the bleaching position in the μs region (Fig. 2g). The time-resolved blue-shift of bleaching signal was also clearly observed by using the ns-transient absorption spectroscopy (See Supplementary Fig. 8). The red-shift of bleaching of the CdS/CuS HNCs in comparison with the CuS NCs is derived from the different LSPR position.

It is noteworthy that a broad and structureless absorption derived from the trapped holes in the CdS phases was observed as positive signal in the visible region^20,21^, providing direct evidence of LSPR-induced hole transfer from the CuS to CdS phase. The blue-shift of bleaching and the absorption of trapped hole of CdS makes significant difference between the CuS NCs and the CdS/CuS HNCs in the transient absorption spectra at the timescale of μs region (see detail explanation in Supplementary Fig. 8). As the decay of the trapped hole corresponds to charge recombination, the charge recombination rate between the CuS phase and CdS phase was estimated to be 1.1 × 10^5^ s^–1^ (Fig. 2e), indicating that the charge separation was long-lived.

**Fig. 3:**
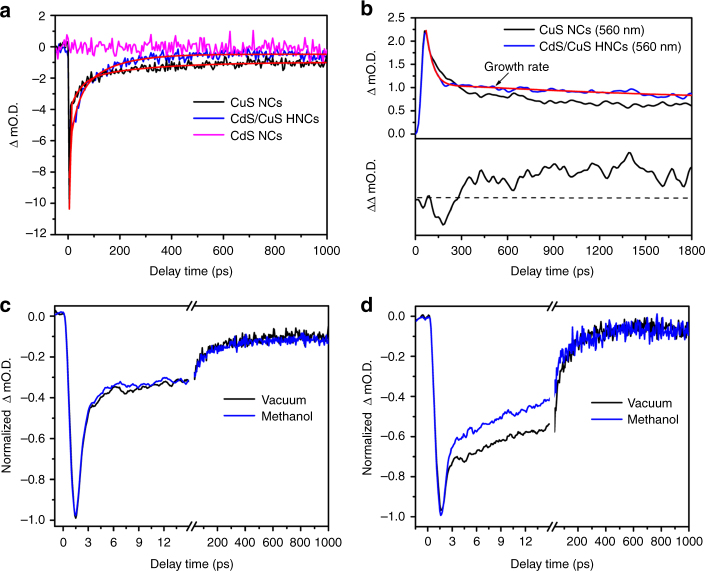
Kinetic profiles and quenching study of nanocrystals after LSPR excitation. **a** Kinetic profiles of CuS nanocrystals (NCs), CdS NCs, and CdS/CuS heterostructured NCs (HNCs) at 1000 nm; red line is the best fit. **b** Decay profiles of CuS NCs and CdS/CuS HNCs at 560 nm; red line is the best fit. The rising component corresponds to the trapped holes in the CdS phase of CdS/CuS HNCs. **c**, **d** Quenching experiments by hole scavenger (methanol vapor: 20 Torr) for **c** CuS NCs and **d** CdS/CuS HNCs probing at 1000 nm. The ΔmO.D. means the change of optical density. Excitation wavelength: 1200 nm

As the hot carrier generated in CuS NCs should decay within a few ps, TA measurement in the ps region is essential for elucidating the hot hole transfer mechanism. Therefore, we measured the TA of the CuS NCs and CdS/CuS HNCs in the ps region to investigate the LSPR-induced hot hole transfer mechanism (Fig. 3a). As the LSPR peak of the CuS phase is sensitive to carrier density^18^, the hole transfer can be determined using the time-resolved change in the LSPR band. As shown in Fig. 3a, the LSPR recovery upon excitation closely resembled the laser pulse shape, followed by a slower recovery. A similar trend was previously observed in the TA measurements of CuS NCs by Cozzolli and coworkers, although slow components were not discussed^11^. The LSPR bleaching and recovery of the CuS NCs at 1000 nm was well-fitted by a triexponential function with two clear components, 0.5 ps (*τ*_1_) and 110 ps (*τ*_2_), and one unclear component, for which the time constant (*τ*_3_) could not be measured in the time frame of fs TR-IR (3 ns) (Supplementary Table 1). Since carrier trapping significantly affects LSPR recovery, we concluded that this multi-step recovery of LSPR reflected carrier-trapping-mediated relaxation, in addition to conventional LSPR decay. As the LSPR-mediated hole-trapping rate of CuS NCs has been reported as < 2 ps, the fastest component corresponded to both ultrafast carrier trapping and a carrier-scattering-mediated decay process^17^. The decay components *τ*_2_ and *τ*_3_ corresponded to the relaxation process through hole trapping in deep and shallow states, as discussed below.

The kinetic trace of the LSPR bleaching and recovery of CdS/CuS HNCs was also well-fitted by a triexponential function with components of 0.4 ps (*τ*_1_), 80 ps (*τ*_2_), and > 3 ns (*τ*_3_). The *τ*_2_ value of the CdS/CuS HNCs was smaller than that of the CuS NCs. We conducted quenching experiments using methanol as a hole scavenger to determine whether hole extraction affected the LSPR recovery. As shown in Fig. 3c, d, in the presence of methanol vapor, a slight increase in the recovery rate was observed for the CuS NCs. This feature was dramatically increased in the CdS/CuS HNCs. This change could be caused by the spectral change in the LSPR band due to the loss of holes. The dramatic increase in the LSPR band recovery rate for CdS/CuS HNCs by methanol reflected charge separation in the HNC, which led to efficient hole scavenging by methanol. Based on these results, it was conceivable that hole extraction from the CuS phase promoted LSPR recovery at 1000 nm. Therefore, we concluded that the smaller *τ*_2_ value of the CdS/CuS HNCs, compared with that of the CuS NCs, reflected hole transfer from the CuS phase to the CdS phase. From the *τ*_2_ values of the CuS NCs and CdS/CuS HNCs, the hole transfer rate (*k*_2_) in the CdS/CuS HNCs was estimated to be 3.4 × 10^9^ s^–1^.

### The plasmon-induced hot hole transfer mechanism

Figure 3b shows the kinetic profiles of CuS NCs and CdS/CuS HNCs at 560 nm, tracing hole trapping in the CdS phase. The decay profile of the CuS NCs was well-fitted by a biexponential decay function. These components have been reported as trapped holes in the CuS NCs^17^. In contrast, the decay profile of CdS/CuS HNCs at 560 nm was not well-fitted by this function, but by a biexponential decay and a single exponential growth function, reflecting the evolution of absorption corresponding to the trapped holes in the CdS phases. As the hole trapping rate in the CdS NCs was faster than 1.4 × 10^12^ s^–1^ (ref. ^21^), the growth rate (*k*_r_ = 5.6 × 10^9^ s^–1^) mainly reflected hole transfer from the CuS phase to the CdS phase (Supplementary Table 1). The agreement of the hole extraction rate from the CuS phase (*k*_2_) with the hole accepting rate of the trapped state (*k*_r_) of the CdS phase strongly supported our hypothesis. The quantum efficiency of observed hole transfer was estimated to be 19% by using the following equation: ${{\Phi }} = \frac{{n_{\mathrm{h}}}}{{N_{{\mathrm{photons}}}}}$, where *n*_h_ is the number of trapped holes generated in the CdS domain, and *N*_photons_ is the number of photons absorbed by the CuS domain (see Supplementary Note 2 for detailed calculation)^21^.

It should be emphasized that the *Φ* of hole transfer is significantly high although hole transfer rate in the present system is much slower than the decay rate of hot holes (approximately 2 × 10^12^ s^–1^). This contradiction strongly suggested that the hot holes were not directly injected into the CdS phase, but transferred stepwise via transit through the carrier trapping state. We have named this carrier transfer process plasmon-induced transit charge-transfer (PITCT). This mechanism was further confirmed by LSPR-induced hole transfer to a hole-scavenging organic molecule (triphenylamine (TPA)). In TPA-protected CuS NCs, hole transfer to TPA was observed at a rate of 3.3 × 10^10^ s^–1^ after excitation of the LSPR band. Furthermore, the slower hole transfer rate to TPA compared with the decay rate of hot holes strongly supported the PITCT mechanism (Supplementary Figs. 9–13).

Furthermore, the theoretical calculation of mean free path of hot holes was conducted to support our hypothesis. By using the jellium model, the mean free path of hot holes in the CuS NCs was estimated to be 2.87 nm (see Supplementary Note 2 for detailed calculation)^22,23^. Taking the decay channel of holes trapping into consideration, the actual mean free path of hot holes should be shorter than the theoretically value estimated using the jellium model. Based on the conventional tunneling mechanism, only the hot holes generated within the region defined by the mean free path from the heterointerface of the CdS/CuS HNCs can participate effectively in interfacial hole transfer. Therefore, it is unlikely that the present hole transfer with an efficient *Φ* proceeds via the conventional mechanism. The short mean free path of hot holes strongly enforces the hole transfer via a PITCT mechanism.

To prove the contribution of PITCT to the IR responsive catalytic activity, we used methylene blue (MB, oxidation potential of 0.523 V vs. NHE^24^) as a probe. The CdS/CuS HNCs exhibited oxidation catalytic activity superior to those of CuS NCs and CdS NCs under NIR light irradiation (Supplementary Fig. 14). The degradation kinetics were pseudo-first-order dynamics, so the reaction rate could be estimated using the following equation: ln(*C*/*C*_0_) = –*n* × *t* + *b*, where, *C*_0_, *C*, *n*, *t* and *b* are the initial MB concentration, the MB concentration in solution, the degradation rate constant of degradation, the reaction time, and the reaction constant, respectively. As shown in Supplementary Fig. 14, the *n* value of CdS/CuS HNCs was 34 times higher than that of pristine CuS NCs due to the efficient hot hole extraction from CuS to the VB of CdS via PITCT, indicating that the PITCT process realized efficient NIR light-responsive catalytic activity.

## Discussion

**Fig. 4:**
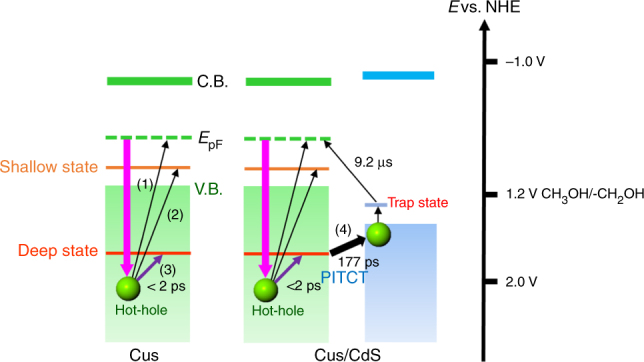
Schematic illustration of LSPR-induced stepwise hole transfer process. Decay processes of hot holes generated in CuS NCs and CdS/CuS HNCs are shown. The red arrows means plasmon excitation by near-infrared (NIR) light. For CuS NCs, the generated hot holes decayed via hole–hole and phonon–hole scattering (1) or ultrafast hole trapping to the shallow (2) or deep trapping state (3), followed by relaxation to the intrinsic hole state. In CdS/CuS heterostructured nanocrystals (HNCs), the holes in the deep trapping state transferred to the valence band (VB) of the CdS phases (4, PITCT) and the holes in the CdS phases moved to the trapping state, showing structureless absorption in the visible region and recombination to the initial state. PITCT: plasmon-induced transit carrier transfer

We have summarized the PITCT mechanism in Fig. 4. The hole transfer rate observed in this experiment (3.4–5.6 × 10^9^ s^–1^) was much slower than the decay rate of hot holes in CuS. This slow PITCT rate realized LSPR-induced hole transfer and a subsequent oxidation reaction using NIR light. We anticipate that PITCT could solve the problems of conventional LSPR-induced energy conversion, such as ultrafast relaxation of hot carriers and energy loss by charge recombination, and facilitate efficient energy conversion using low-energy IR light with plasmonic materials. Furthermore, the suitable band alignments and the defect sites play an important role for the long-lived charge separation with high quantum yields. PITCT adds a new dimension to optical materials science by controlling LSPR-induced carrier dynamics through defect engineering technology.

## Methods

### Synthesis of hexagonal plate-shaped *cv*-CuS NCs

A mixture of copper (I) acetate (0.123 g, 1 mmol), and oleylamine (10 mL) was degassed at 160 °C for 30 min. A solution of sulfur (0.048 g, 1.5 mmol) in 1-octadecene (15 mL) was injected rapidly into the mixture under a nitrogen atmosphere, and stirred for 10 min. After the reaction, the resulting product was purified by adding ethanol–hexane (*v*:*v* = 1:1) mixed solvent to the solution, centrifuging twice, and then redispersed the precipitate in hexane.

### Synthesis of CdS/CuS HNCs

The metal thiocarbamate precursor of the hole acceptor phase (CdS) was prepared as follows: Sodium diethyldithiocarbamate (5 g, 22 mmol) was dissolved in water (100 mL) and a solution of Cd(NO_3_)_2_**·**4H_2_O (3.085 g, 10 mmol) in water (100 mL) was added, followed by stirring for 1 h. The resulting product was washed twice with water, centrifuged, washed again by ethanol, centrifuged and dried in an oven at 70 °C for 12 h to obtain the Cd precursor. A mixed solution of CuS NCs (0.2 mmol), oleylamine (5 mL), and 1-octadecene (10 mL) was degassed at 150 °C for 30 min. A solution of the Cd precursor (50 mg) in oleylamine (2 mL) was prepared with ultrasonication and injected into the above mixed solution under a nitrogen atmosphere at a rate of 0.1 mL min^−1^, and stirred for a further 30 min after finishing the injection. The product was purified by centrifugation with hexane–ethanol (*v*:*v* = 1:1) mixed solvent, and redispersed in hexane.

### Characterization

Transmission electron microscopy (TEM) and high resolution TEM (HRTEM) characterizations were performed on JEM1011 (JEOL) and JEOL-2200FS (equipped with Cs-corrector for TEM) electron microscopes with operating voltages of 100 kV and 200 kV, respectively. HAADF-STEM and EDS-mapping were performed on a JEM-ARM200F (spherical aberration correction device) electron microscope with an operating voltage of 200 kV. The XRD patterns were recorded on a PANalytical X’Pert Pro MPD diffractometer, with Cu Kα radiation (*λ* = 1.542 Å) at 45 kV and 40 mA. Ultraviolet–visible–near–infrared (UV–vis–NIR) absorption spectra were recorded using a U-4100 spectrophotometer (Hitachi). X-ray fluorescence (XRF) spectroscopy elemental analysis was carried out using Element Analyzer JSX-3202C (JEOL). ^1^H NMR spectra were measured on JEOL JNMECP300 (300 MHz) spectrometers. Matrix-assisted laser desorption ionization–time-of-flight mass spectrometry (MALDI-TOF MS) was performed on a Bruker Autoflex Speed instrument using *trans*-2-[3-(4-*tert*-butylphenyl)-2-methyl-2-propenylidene] malononitrile (DCTB) as the matrix.

### Photocatalytic degradation of methylene blue

The photocatalytic activities of CuS NCs and CdS/CuS HNCs were evaluated using an NIR light source (NIR light power density, 40 mW cm^–2^). To fabricate the NIR-light, a 300-W Xe light source (Cermax, Excelitas Technology) was irradiated through broadband dielectric mirrors (region, 750–1100 nm, R300-32J, THORLABS) to cut out light of 750–1100 nm. Water-soluble NCs (0.05 mmol) were suspended in water (3 mL) containing methylene blue (MB, 90 mg), and the suspension was sealed in a quartz cell. After different reaction times, a 0.6-mL aliquot of the suspension was sampled into a plastic tube and centrifuged to remove the NCs. The remaining MB concentration was determined by the characteristic MB wavelength at 464 nm using UV–vis–NIR spectrophotometry.

### Transient absorption measurements

The behavior of NIR-LSPR-generated holes was investigated using homemade femtosecond-to-second time-resolved spectrometers^25^. In the femtosecond-to-nanosecond region, experiments were performed using a conventional pump–probe technique based on a Ti–sapphire laser system (Spectra Physics, Solstice and TOPAS Prime; duration, 90 fs; wavelength, 800 nm; repetition rate, 1 kHz). In this experiment, a 1200-nm laser pulse was used as the pump pulse. For the microsecond measurements, in the visible-to-NIR region, a halogen lamp (50 W) and an InGaAs detector were used as the light source and detector, respectively. Transient absorption spectra were measured from 500 nm to 1 μm. The spectra were obtained at 200-nm intervals and averaged over 300 scans for a spectrum by irradiating the sample using a pumping wavelength of 1200 nm generated by UV laser pulse from a Nd:YAG laser (Continuum, Surelite-II; duration, 6 ns; wavelength, 355 nm; repetition rate, 0.01–5 Hz). The powdery sample was fixed on a CaF_2_ plate with a density of ∼1 mg cm^–2^, and the sample plate was placed in a stainless-steel cell. Measurements were performed under vacuum at room temperature. For the ns-transient absorption measurements, the randomly-interleaved-pulse-train (RIPT) method was employed^26^. The pump-pulse and probe source is a picosecond laser, PL2210A (EKSPLA, 1 kHz, 25 ps, 355 nm, 0.3 mJ), and a supercontinuum (SC) radiation source (SC-450, Fianium, 20 MHz, 50–100 ps pulse width depending on the wavelength, 450–2000 nm), respectively. A 1064 nm-laser pulse was selected to excite the plasmon response. Generally, a chloroform solution of as-obtained samples in a 10 mm-thick quartz cell under vigorously stirred was performed to do the ns-TA measurements at room temperature.

### Data availability

The data sets within the article and Supplementary Information of the current study are available from the authors upon request.

## Electronic supplementary material

Supplementary Information
