# Supplementary material for: Near infrared light induced plasmonic hot hole transfer at a nano-heterointerface
Source: Nat Commun. 2018 Jun 13;9:2314. doi: 10.1038/s41467-018-04630-w (PMC5997981; doi:10.1038/s41467-018-04630-w)
Supplement: Supplementary file 1 — Supplementary Information [file 41467_2018_4630_MOESM1_ESM.pdf]

Supplementary Information

Near Infrared Light Induced Plasmonic Hot Hole Transfer at a Nano-Heterointerface

Z. Lian et al.

Supplementary Note 1

Synthesis of 4-nm *w*-CdS NCs

The CdS NCs were synthesized according to a previous report with a slight modification[1]. Briefly, cadmium stearate (0.227 g, 0.3 mmol), *N*,*N*'-dibutylthiourea (0.056 g, 0.3 mmol), oleylamine (0.99 mL, 3.0 mmol), and di-*n*-octylether (9 mL) were added to a three-necked flask (100 mL), heated to 260 °C at a rate of 20 °C min^{-1} under N_2, and then stirred for 20 min. The product was purified by centrifugation with ethanol, and the precipitate was redispersed in hexane.

Investigation of CdS/CuS HNCs formation process

The formation mechanism of the CdS/CuS HNCs was investigated by controlling the growth of the CdS phases. The CdS/CuS HNCs were synthesized using a seeded growth method, as shown in Supplementary Figure 2. First, plate-shaped CuS NCs were synthesized using a hot injection method, as outlined above. The Cd thiocarbamate precursors were decomposed to give CuS NCs with multiple CdS phases.

To confirm our hypothesis that in-situ decomposition of the Cd carbamate precursor occurred on the surface of the CuS NCs, several controlled experiments were performed. When decomposition of the Cd carbamate precursor was conducted in the absence of CuS NCs, only tetrapod-shaped CdS NCs were obtained, as shown in Supplementary Figure 3. Supplementary Figure 4 shows that, in the presence of CuS NCs, an increase in reaction time afforded larger wurtzite CdS phases deposited on the CuS surface. Furthermore, the LSPR peak was red-shifted by increasing CdS size due to the change of dielectric environment of CuS phases, which were now occupied by large CdS phases in the HNCs. When a large amount of Cd precursor was used, numerous wurtzite CdS phases with high densities were deposited on the surface of the CuS NCs, as shown in Supplementary Figure 5. The LSPR peak was red-shifted due to the change of dielectric environment through high-density CdS. Therefore, it was concluded that the CdS phases were deposited and epitaxially grown on the CuS surface as indicated by HRTEM analysis. This proposed route could be used to design proper functional materials for constructing NIR energy conversion systems.

Preparation of water-soluble CuS NCs and CdS/CuS HNCs

The synthesized CuS NCs and CdS/CuS HNCs were transferred from a chloroform phase to an aqueous phase using an 11-mercaptoundecanoic acid (MUA)-assisted phase transfer method. A chloroform solution of MUA (40 mL, 50 mM) was added to a chloroform solution (10 mL) of CuS NCs or CdS/CuS HNCs (0.2 mmol) under N_2 and the mixture was vigorously stirred for 30 min. The MUA-capped CuS NCs or CdS/CuS HNCs were collected by centrifugation, redispersed in 0.1 M NaOH aqueous solution (10 mL), and centrifuged again. Finally, the as-obtained samples were dispersed in water (10 mL).

Synthesis of 4-diphenylaminobenzenethiol (TPA-SH)

4-Bromotriphenylamine (6.189 mmol, 2.0066 g) was dissolved in dry THF (20 mL) and cooled to –78 °C under N_2. A hexane solution of *n*-BuLi (1.6 M, 10 mL) was added to the solution and stirred under N_2 for 30 min, before adding sulfur powder (9.297 mmol, 297.5 mg). After stirring for 30 min, AcCl (12.4 mmol, 0.9 mL) was added to the mixed solution, and the temperature was slowly raised to room temperature. The solution was then stirred overnight under N_2. After the reaction, CH_2Cl_2 (100 mL) was added to the mixture, which was then washed with H_2O. The obtained solution was removed by evaporation and the residue was purified by silica gel column chromatography using a mixture of CH_2Cl_2 and hexane (1:1) as the eluent. The obtained solid (453.1 mg) was dissolved in dry THF and aqueous ammonium solution (28%, 3 mL) was added. The solution was stirred under N_2 for 12 h, then CH_2Cl_2 (100 mL) was added and the solution was washed with H_2O. The solvent was removed by evaporation and the obtained residue was dissolved in CH_2Cl_2. Zn (57.98 mmol, 3.7909 g) and AcOH (2 mL) were added to the solution, followed by stirring under N_2 for 2 h. The obtained solution was filtered using a silica gel column and evaporated to obtain TPA-SH as yellowish solid.

Yield: 16%, 1H NMR (300 MHz, $CDCl_3$): δ 7.24 (t, J = 7.95 Hz, 4H), 7.18 (d, J = 8.7 Hz, 2H), 7.08–7.01 (m, 6H), 6.76 (d, J = 9.0 Hz, 2H), 3.92 (s, 1H).

MALDI-TOF MS: *m/z* 277 [M^+]

Synthesis of triphenylamine derivative-protected CuS

A chloroform solution of CuS (1 mL, 0.1 M) was added to TPA-SH (3 mg) in chloroform (10 mL) and stirred for 1 h. The precipitate was collected by centrifugation and dried under vacuum.

Observation of cation radicals of TPA

A solution of $Cu(ClO_4)_2$ in acetonitrile (20 mL, 0.15 M) was added to a solution of TPA-SH in acetonitrile (5 mL, 0.1 M), and aliquots of equal volume were taken to measure spectra at different times to observe the formation of cation radicals.

Supplementary Note 2

Electrochemical measurements

Mott–Schottky plots: Electrochemical measurements were carried out in a conventional three-electrode single-compartment quartz cell on an electrochemical station (ALS CHI 620C). The working electrodes were prepared as follows: a 5% Nafion solution in lower aliphatic alcohols and water (10 μL) was added to a solution *w*-CdS NCs in hexane (490 μL) and sonicated to obtain a homogeneous solution. Samples (10 μL) were dropped on the glassy carbon (GC) electrode (diameter, 5 mm; area (A), 20 mm^2) and dried in air. A silver chloride electrode (Ag/AgCl) and platinum wire electrode were used as reference and counter electrodes, respectively. A 0.2 M Na_2SO_4 aqueous solution (pH 7.0) was used as an electrolyte. The Mott–Schottky plots were obtained at a fixed frequency of 1 kHz to determine the flat-band potential and carrier density. The potential vs. NHE (normal hydrogen electrode) was calculated using the following equation (Supplementary Equation 1):

$$E_{\mathrm{vs.RHE}} = E_{\mathrm{vs.Ag/AgCl}} + 0.1976 + 0.059 \times \mathrm{pH} \quad (1)$$

Differential pulse voltammetry: Potential steps were applied using the electrochemical station. A platinum electrode was used as the working electrode, along with a Ag/AgCl pseudo-reference electrode and a platinum-disk counter electrode. The electrodes were positioned in a small glass vessel and used as a standard three-electrode cell for differential pulse voltammetry (DPV) measurements to determine the HOMO level of TPA. All samples were purged with argon gas for at least 15 min prior to experiments. The solvent and electrolyte were *N*,*N'*-dimethylformamide (DMF) and tetrabutylammonium hexafluorophosphate ($TBAPF_6$, 0.1 M), respectively. The TPA concentration was adjusted to 1 mM for measurements. The pseudo-reference electrode was calibrated against a ferrocenium/ferrocene (Fc^+/Fc) standard before and after the experiments, and was converted into a normal hydrogen electrode (NHE) with a Fc^+/Fc half-wave potential of 0.63 V vs. NHE[2,3].

Fitting of kinetic profiles

The profiles monitoring LSPR bleaching and recovery of samples in Figures 2b and 3a were fitted to a triexponential growth function (Supplementary Equation 2):

$$y = y_0 - A_1 \exp(-\frac{x - x_0}{\tau_1}) - A_2 \exp(-\frac{x - x_0}{\tau_2}) - A_3 \exp(-\frac{x - x_0}{\tau_3}) \quad (2)$$

where, τ_1, τ_2, and τ_3 are the decay constants, A_1, A_2, and A_3 are amplitudes, x_0 is the center position, and y_0 is the offset. The decay constants obtained from Figures 2b and 3a are listed in Supplementary Table 1.

We employed Supplementary Equation (3) to analyze the decay profile of CdS/CuS HNCs in Figure 3b to estimate the growth rate corresponding to stepwise hole transfer from the surface state of CuS to the CdS phase.

$$y = y_0 - A_1 \exp(-\frac{x - x_0}{\tau_1}) - A_2 \exp(-\frac{x - x_0}{\tau_2}) + A_3 \exp(-\frac{x - x_0}{\tau_3}) \quad (3)$$

The decay constants obtained from Figure 3b are listed in Supplementary Table 1.

Calculation of flat band potentials and band gap structures

Capacitance measurements were performed on the electrode/electrolyte according to the Mott–Schottky equation[4,5]:

For *n*-type semiconducting CdS NCs:

$$\frac{1}{C^2} = \frac{2}{N_D e \varepsilon \varepsilon_0}(E - E_{FB} - \frac{kT}{e}) \quad (4)$$

where C is the space charge capacitance in semiconductor, N_D is the electron carrier density, e is the elemental charge, ε_0 is the permittivity of vacuum, ε is the relative permittivity of the semiconductor, E is the applied potential, E_{FB} is the flat band potential, T is the temperature, and k is the Boltzmann constant.

Supplementary Figure 7 shows the Mott–Schottky plots of $1/C^2$ as a function of the applied potential, from which a positive slope (line) was observed, indicating *n*-type semiconductor. Furthermore, the plots were extrapolated to $1/C^2 = 0$ to determine E_{FB} values of −0.78 V for the CdS NCs. Furthermore, the carrier density N_D can also be calculated using Supplementary Equation 5:

$$N_D = \frac{2}{e \varepsilon \varepsilon_0}\left(\frac{dE}{d(\frac{1}{C^2})}\right) \quad (5)$$

Using the following values, $e = 1.6 \times 10^{-19}$ C, $\varepsilon_0 = 8.86 \times 10^{-12}$ F/m, $\varepsilon = 2.529^2$ for CdS, the N_D value

of the CdS NCs were determined as 6.2 × 10^{15} cm^{-3}. Generally, N_V is the effective density of states (typically ~10^{19}) at the valence band edge. According the following Supplementary Equation (6):

$$E_{vn} = V_{FB} + \frac{kT}{e}\ln(N_D / N_V) \quad (6)$$

Using Supplementary Equation (3), we determined the conduction band edge energy, E_{vn}, was –0.97 V for the CdS NCs[6]. For metallic-like CuS NCs, the valence band edge and Fermi level were reported to be 0.94 V (ref. 7) and 0.80 V (ref. 8). The bandgap energy was 1.90 eV from the estimation of Supplementary Figure 7c, so the conduction band of CuS NCs was -0.96 eV.

Calculation of quantum yield (QY) for PITCT

Sample thickness, L: Firstly, in the CdS/CuS HNCs, the ratio of Cd/Cu was 38/62. The mass ratio of ligands and HNCs was 22.12:77.88 from elemental analysis. Therefore, ρ_{CdS} = 4.82 g cm^{-3}, ρ_{CuS} = 4.60 g cm^{-3}, and $\rho_{Oleylamine}$ = 0.830 g cm^{-3}. The CaF_2 plate was 16 Φ × 1 mm, and the plate density covered by sample was ~1 mg cm^{-2}. The volume (V_1) of CdS/CuS HNCs was calculated using Supplementary Equation (7):

$$V_1 = V_{CdS} + V_{CuS} = \frac{m_{CdS}}{\rho_{CdS}} + \frac{m_{CuS}}{\rho_{CuS}} = \frac{2mg*77.88\%*0.38}{4.82g/cm^3} + \frac{2mg*77.88\%*0.62}{4.6g/cm^3} = 3.3\times10^{-10}\,\mathrm{m}^3 \quad (7)$$

The volume (V_2) of ligands was calculated using Supplementary Equation (8):

$$V_2 = \frac{m_{ligands}}{\rho_{ligands}} = \frac{2mg*22.12\%}{0.83g/cm^3} = 5.33\times10^{-10}\,\mathrm{m}^3 \quad (8)$$

The area (A') of the CaF_2 plate was calculated using Supplementary Equation (9):

$$A' = \pi r^2 = 3.14*(\frac{16}{2})^2\ \mathrm{mm}^2 = 2.00\times10^{-4}\,\mathrm{m}^2 \quad (9)$$

Therefore, L_1, the optical length of CdS/CuS HNCs, and L_2, the length of ligand occupation were calculated using Supplementary Equations (10) and (11), respectively:

$$L_1 = \frac{V_1}{A'} = \frac{3.33\times10^{-10}m^3}{2\times10^{-4}m^2} = 1.67\times10^{-6}\,\mathrm{m} \quad (10)$$

$$L_2 = \frac{V_2}{A'} = \frac{5.33\times10^{-10}m^3}{2.00\times10^{-4}m^2} = 2.67\times10^{-6}\,\mathrm{m} \quad (11)$$

$$L = L_1 + L_2 = 1.67\times10^{-6}m + 2.67\times10^{-6}m = 4.34\times10^{-6}\,\mathrm{m} \quad (12)$$

The quantum yield (Φ) of PITCT was estimated using Supplementary Equation (13):

$$\Phi = \frac{n_\mathrm{h}}{N_\mathrm{photons}} \tag{13}$$

where n_h is the number of trapped holes generated in the CdS domain, and N_photons is the number of photons absorbed by the CuS domain.

N_photons was estimated using Supplementary Equation (16):

$$N_\mathrm{photons} \approx \frac{A \times \text{Total energy of one pulse}}{\text{Energy of single photon}} \cdot (1\text{-}10^{\text{-O.D.1200}}) \tag{14}$$

where A is a ratio of beam intensity within the FWHM of laser pulse (0.5) to the whole intensity. The total energy of one pulse was 6 μJ, and the energy of single photon was estimated using Supplementary Equation (15):

$$\text{Energy of single photon} = \frac{hc}{\lambda} \tag{15}$$

where h, c, and λ are Planck's constant (6.63×10^{-34} J·s), the speed of light (3.00×10^{8} m·s^{-1}) and the laser wavelength (1200 nm), respectively. From Supplementary Equation (15), the energy of a single photon was calculated as 1.66×10^{-19} J. Therefore, the photon number per pulse was estimated to be 3.61×10^{13}. The volume of the pump laser path in the sample pellet was calculated to be 2.80×10^{-7} cm^3 from the FWHM of the pump laser (287 μm) and optical path length (L, 4.34 μm). The O.D. of the sample was 1.0 at the excitation wavelength (1200 nm). Therefore, N_photons was calculated as 5.81×10^{19} cm^{-3}. The absorption coefficient, α (cm^{-1}), was estimated using Supplementary Equation (16):

$$10^{\Delta\Delta\text{O.D.1200nm}} = e^{-\alpha L} \tag{16}$$

where L is the optical path length of the pellet and ΔΔO.D.@560 nm is 0.00035. From Supplementary Equation (16), α was estimated to be 1.90 cm^{-1}. As the absorption cross-section of the free carrier is expressed by $\sigma = \alpha/n_\mathrm{h}$, n_h was expressed by Supplementary Equation (17):

$$n_\mathrm{h} = \frac{\alpha}{\sigma} \tag{17}$$

where σ is the absorption cross-section of a trapped hole in CdS NCs (1.74×10^{-19} cm^2) from the absorption coefficient of a trapped hole in CdS NCs (ε), which was estimated from the relationship between the absorption of a trapped hole in CdS nanorods and cation radicals of phenothiazine (ref. 21), and Supplementary Equation (18):

$$\sigma = \frac{\varepsilon}{N_\mathrm{a}} \tag{18}$$

where N_a is the number density of CdS and ε is the absorption coefficient of a trapped hole in CdS.

The n_h value was calculated as 1.09×10^{19} cm^{-3}. Finally, the Φ of PITCT was estimated to be 19% using Supplementary Equation (13).

Estimation of HOMO level of TPA

We employed DPV to estimate the position of the highest occupied molecular orbital (HOMO) level[9]. As shown in Supplementary Figure 11, the first oxidation and reduction peak were clearly identified. From the redox peaks, the HOMO level position in TPA-SH was estimated to be 0.98 V (vs. NHE).

Absorption spectrum of TPA radical cation

The absorption spectrum of the TPA radical cation ($TPA^{\bullet+}$) was measured by adding the oxidative reagent (Cu^{2+}) to the acetonitrile solution of TPA. After addition the oxidative reagent, new absorption peaks at 785 nm was observed, as shown in Supplementary Figure 13a. The absorption decreased with an increase of broad absorption peaks in the 400–580 nm and 1000–1600 nm regions. The absorption peak at 780 nm was derived from $TPA^{\bullet+}$, while the absorption peaks at 476 nm and 1400 nm were assigned to the TPA dimer[2,10].

TA measurements and TPA-protected CuS

For further confirmation of LSPR-induced hole transfer from CuS NCs, we conducted fs-TA absorption measurements of TPA-protected CuS (Supplementary Figures. 9 and 10). Generally, radical cations of molecules show clear absorption compared with the trapped hole in the CdS domain. The HOMO level of TPA was positioned between the Fermi level of CuS and valence band of CdS, which is suitable for LSPR-induced hole transfer from CuS. The HOMO level of TPA-SH (0.98 V) was determined using electrochemical techniques, as shown in Supplementary Figures. 11 and 12. The decay profiles at 750 nm for TPA-coordinated CuS NCs are shown in Supplementary Figure 13b. As expected, after excitation of the LSPR band using a 1200-nm laser, growth in the absorption derived from $TPA^{\bullet+}$ was observed. It should be emphasized that the growth rate of $TPA^{\bullet+}$ (3.3×10^{10} s^{-1}) was significantly slower than the hot hole decay rate (5×10^{12} s^{-1}). If the hot hole transfer proceeded using the conventional carrier tunneling mechanism, the hole transfer rate would be comparable or faster than the hot hole decay rate in CuS. Therefore, we concluded that the hole transfer in the present system proceeded stepwise. This evidence strongly indicated that hole transfer proceeded not via a tunneling-

based hot hole transfer mechanism, but via PITCT in the present system.

Theoretical calculation of mean free path of hot holes in CuS NCs

The mean free path of hot holes generated in CuS NCs was calculated by using the jellium model[11,12]. Based on the treatment by J. J. Quinn and R. A. Ferrell[11,12], the lifetime of hot carrier is expressed by following Supplementary Equation (19),

$$\tau = 263\, r_s^{-5/2}(E - E_F)^{-2} \quad \text{fs eV}^2 \tag{19}$$

where r_s is the dimensionless carier-density parameter and E-E_F is the excitation energy of the hot carruer with respect to the Fermi level E_F. Since the excitaion energy of hot hole is equal to the energy of irradiated light, E-E_F was set to 1.00 eV. The r_s value can be estimated by Supplementary Equation (20),

$$r_s = \frac{\left(\frac{3}{4\pi n}\right)^{1/3}}{a_0} \tag{20}$$

where a_0 is the Bohr radius. The carrier density n of CuS has been reported to be 0.98×10^{22} cm^{-3} from the value estimated by discrete dipole approximation[13]. By using the n value, the r_s value was calculated to be 5.48. The lifetime of hot hole in CuS was estimated to be 3.74 fs from Supplementary Equation (19). The mean free path of hot hole can be estimated to be 2.87 nm by the following Supplementary Equation (21).

$$\lambda = \tau v_F \tag{21}$$

where v_F is the Fermi velocity calculated using the r_s value[14].

As we discussed in the manuscript, some hot hole trapping process can reduce the lifetime of the hot holes. Thus, the actual mean free path of hot holes should be shorter than the calculated value.

NIR-light induced photocatalytic pollutant degradation using CuS NCs and CdS/CuS HNCs

The NIR-light-induced photocatalytic activities of the CdS/CuS HNCs for pollutant degradation were evaluated, as shown in Supplementary Figure 14. Methylene blue (MB) degradation was chosen as the probe reaction because the oxidation potential of MB was 0.523 V vs. NHE. The catalytic activities of CuS and CdS NCs were examined under identical reaction conditions for comparison. The CdS/CuS HNCs exhibited superior activity compared with the other NCs due to highly efficient charge

separation, as shown in Supplementary Figure 14a. The degradation kinetics were pseudo-first-order dynamics, so the reaction rate could be estimated using the following equation: $\ln(C/C_0) = -n \times t + b$, where, C_0, C, n, t, and b are the initial MB concentration, the MB concentration in solution, the degradation rate constant of degradation, the reaction time, and the reaction constant, respectively. As shown in Supplementary Figure 14b, the *n* value of CdS/CuS HNCs was 34 times higher than that of pristine CuS NCs due to the efficient hot hole extraction from CuS to the VB of CdS via PITCT (Figure 4). Thermal degradation of MB was ruled out because no degradation was observed in the absence of NCs. The formation of superoxide anions ($O_2^{\cdot-}$), an important active radical for photocatalytic reactions, is known to result from the reaction between dissolved oxygen in the reaction system and photogenerated electrons. Therefore, we carried out a similar reaction in the absence of oxygen as a negative control experiment. By purging with Ar gas to remove oxygen, the formation of superoxide anions could be inhibited. Even in Ar-purged solution, the MB degradation ratio only slightly decreased from 50% to 47% after reacting for 1 h under NIR light irradiation in the CdS/CuS HNCs. These results indicated that $O_2^{\cdot-}$ was not a key factor affecting the photocatalytic performance of CdS/CuS HNCs. Therefore, the key factor affecting the photocatalytic activity was plasmon-induced hole extraction from the CuS to CdS phase through the PITCT pathway.

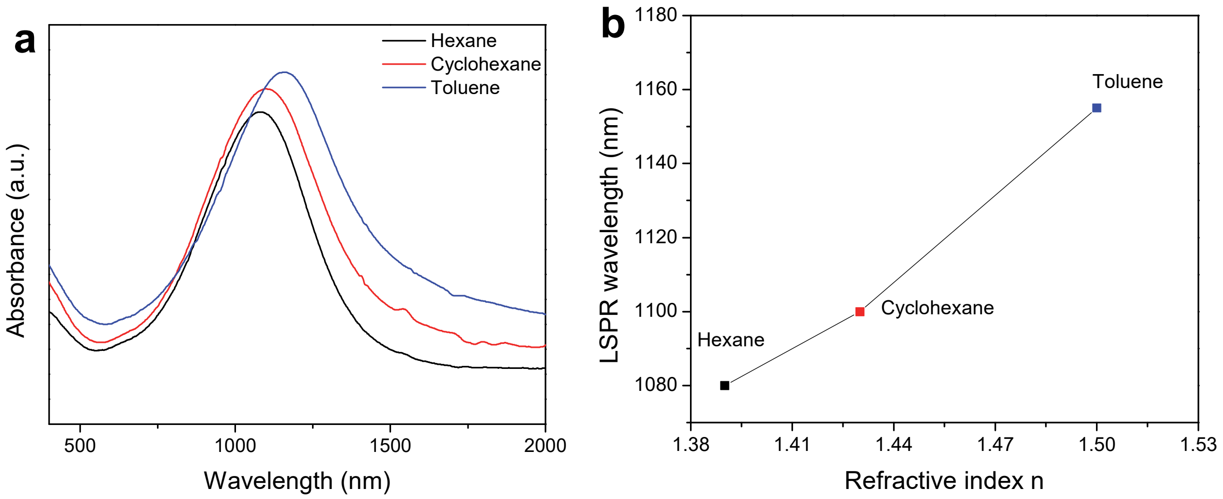

Supplementary Figure 1. The solvent effect of plasmon materials. a, UV–vis–NIR spectra of CuS NCs in different solvents. **b,** LSPR wavelength as a function of the refractive index of the solvent. For plasmon materials, the LSPR peak is affected by the refractive index (n) of solvents. Therefore, the LSPR peak was red-shifted by an increasing n value.

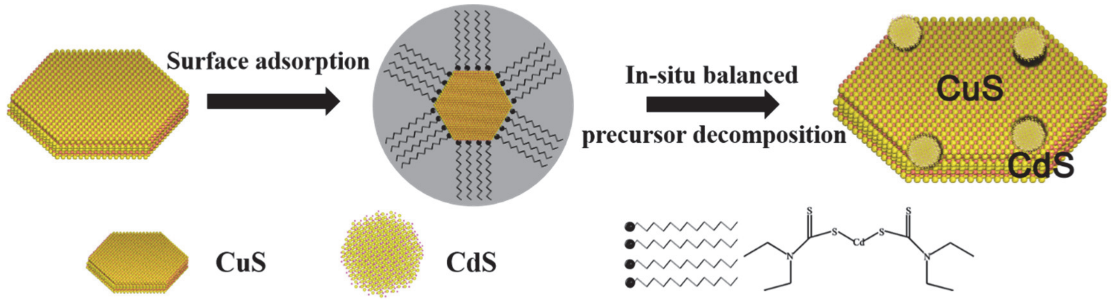

Supplementary Figure 2. Illustration of formation processes. Schematic illustration of the CdS/CuS HNCs formation process.

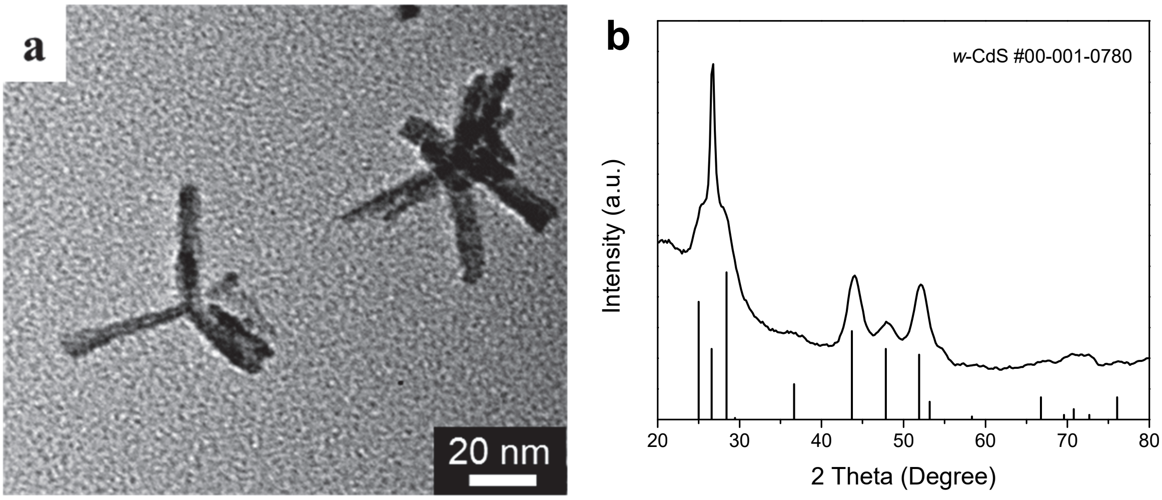

Supplementary Figure 3. Characterization of the CdS NCs. a, TEM image and **b**, XRD pattern of CdS tetrapod obtained with the absence of CuS nanodisk in the synthesis under the same reaction conditions.

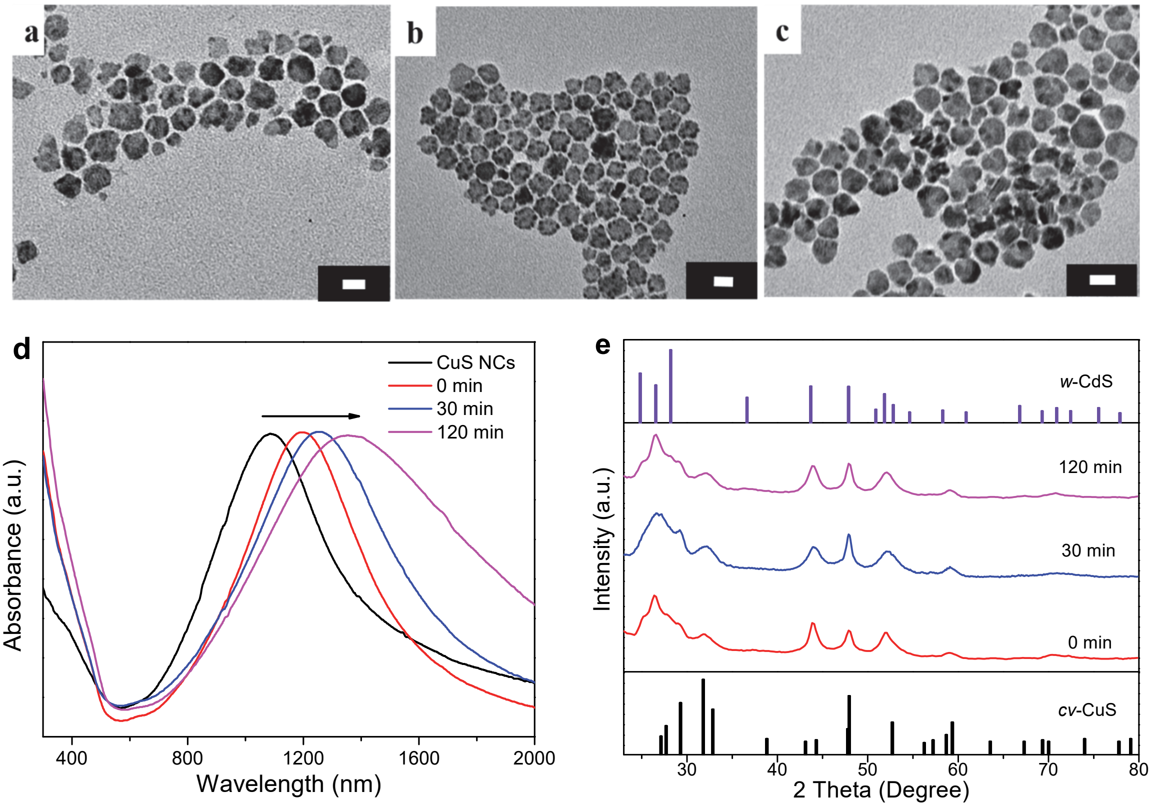

Supplementary Figure 4. Characterization of different reaction time in the synthetic processes of CdS/CuS HNCs. a–c, TEM images of CdS/CuS HNCs with different reaction time, **d,** absorption spectra, and **e,** XRD patterns of CdS/CuS HNCs synthesized with different reaction times: (**a**) 0 min (CdS size, 2.0±0.6 nm), (**b**) 30 min (CdS size, 3.8±0.8 nm), (**c**) 120 min (CdS size, 5.7±1.5 nm). Scale bars: 20 nm.

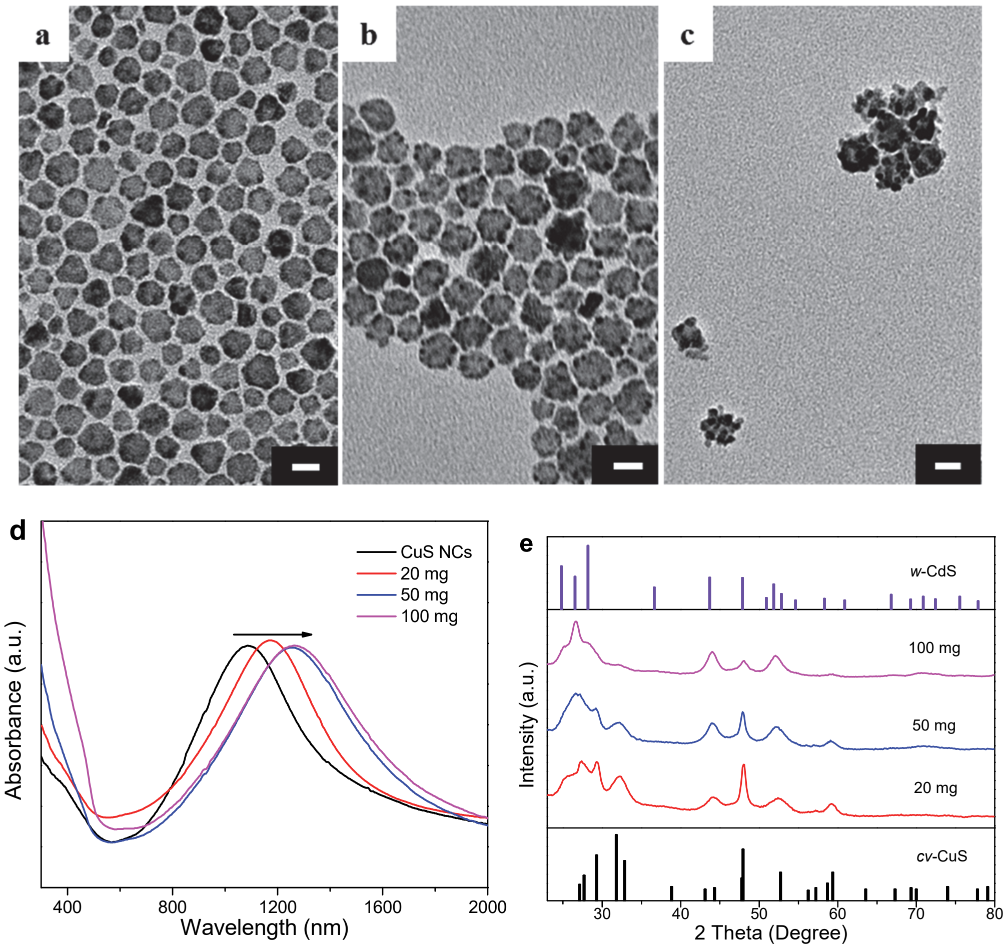

Supplementary Figure 5. Characterization of CdS/CuS HNCs synthesized under the different amount of CdS precursors. TEM images, absorption spectra and XRD patterns of different quantity of Cd precursors, **a**, 20 mg, **b**, 50 mg, **c**, 100 mg; **d**, absorption spectra and **e**, XRD patterns of the CuS and other heterostructure with different usage of Cd precursors. Scale bars, 20 nm.

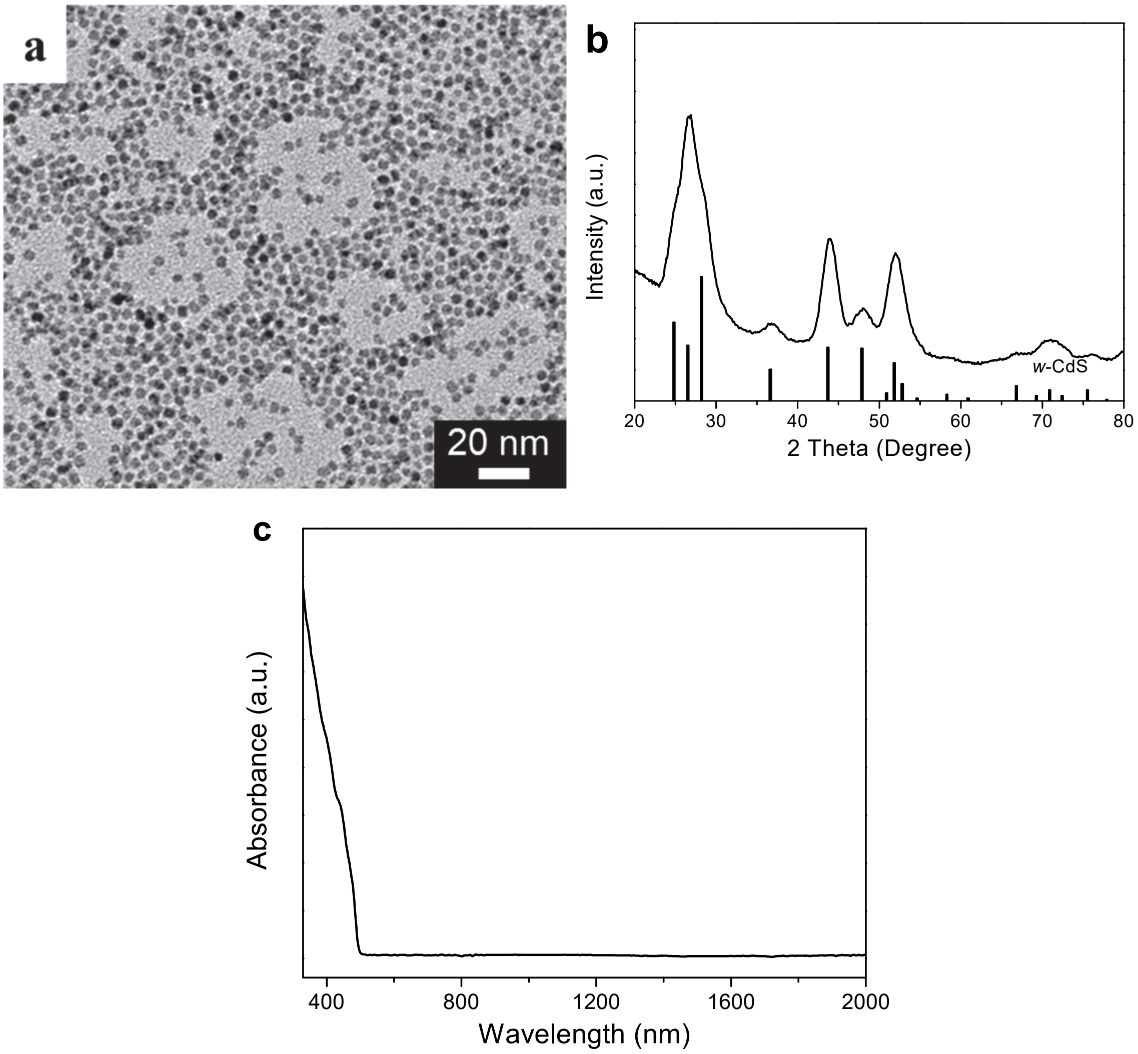

Supplementary Figure 6. Characterization of *w*-CdS NCs. a, TEM image (average diameter, 4.1±0.7 nm), **b,** XRD pattern, and **c,** UV–Vis–NIR absorption spectrum of *w*-CdS NCs.

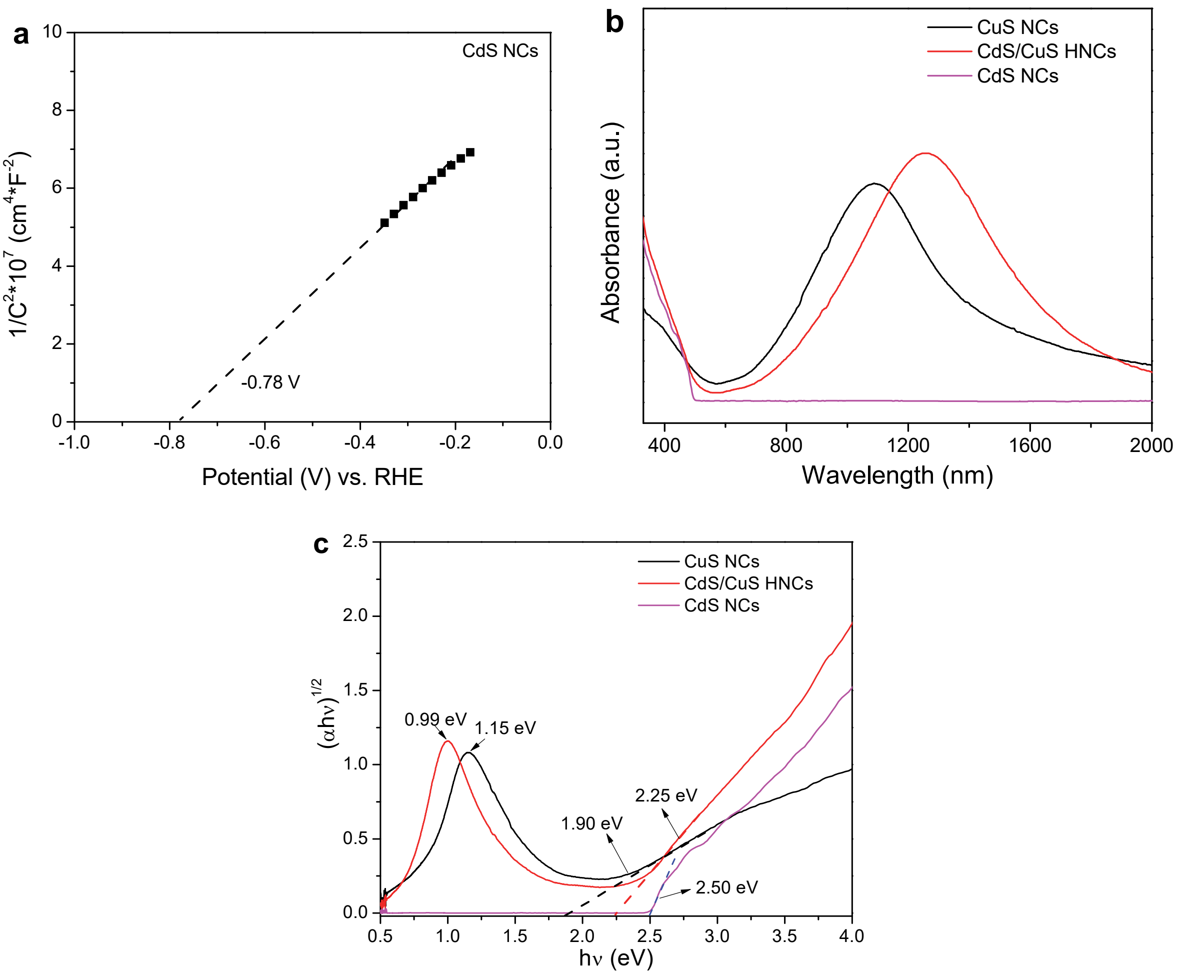

Supplementary Figure 7. Electrochemical testing and optical property of CdS NCs, CdS NCs and Cds/CuS HNCs. a, Mott–Schottky plots of as-obtained CdS NCs, **b,** Absorption spectra of CuS NCs, CdS NCs and CdS/CuS HNCs, **c,** Tauc plots to estimate bandgaps of CdS NCs and CuS NCs.

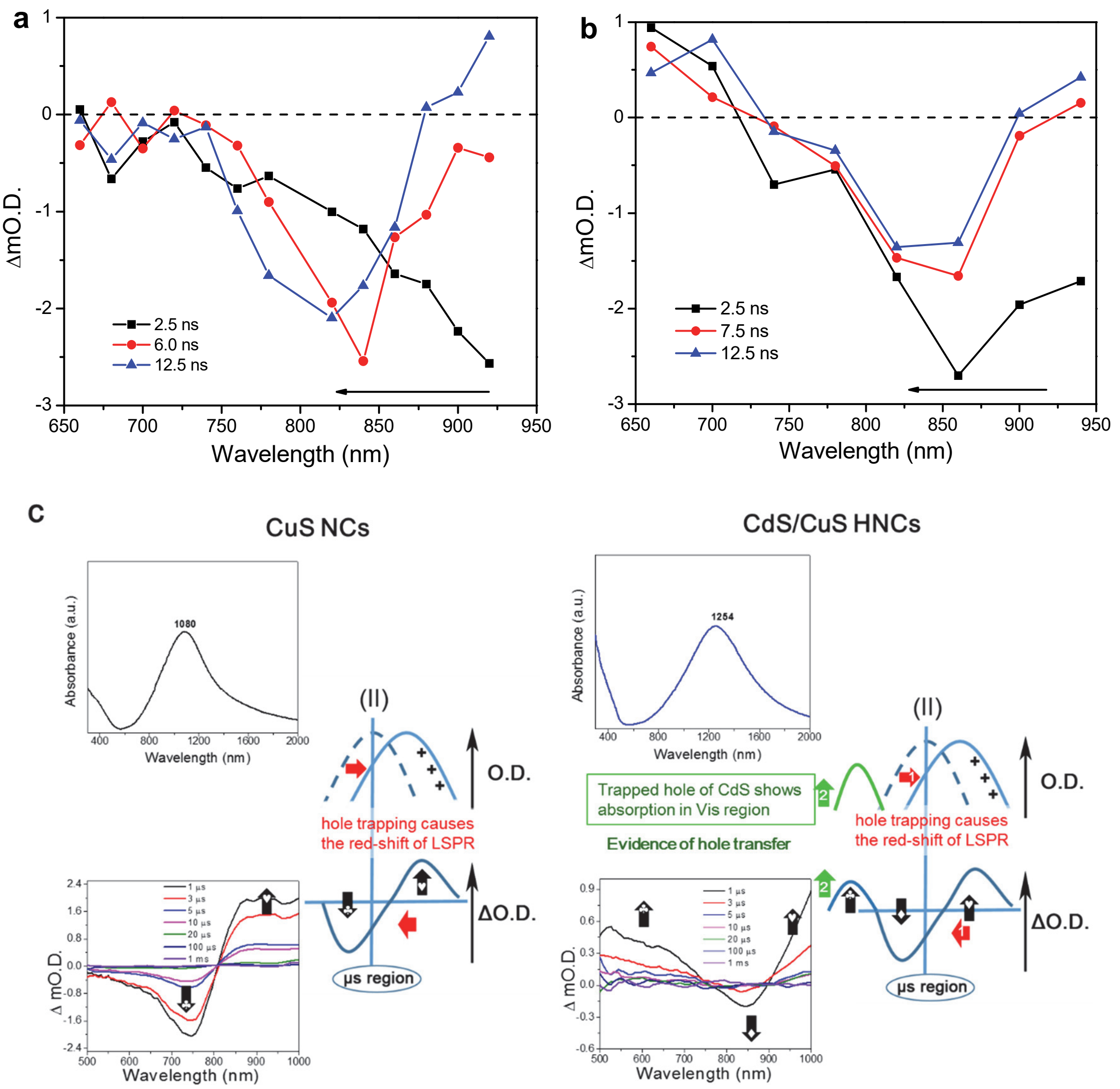

Supplementary Figure 8. Transient absorption spectra in the nanosecond region and detailed schematic illustration for explanation of time-resolved change of transient absorption. Time-dependent transient absorption spectral changes for **a,** CuS NC and **b,** CdS/CuS HNCs at 1064-nm laser excitation in the ns region. **c,** Detailed explanation of time-resolved spectral shift in μs-region.

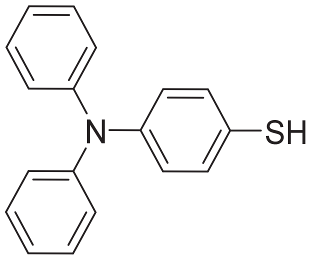

Supplementary Figure 9. The molecule structure of the triphenylamine derivative. Molecular structure of 4-diphenylaminobenzenethiol (TPA-SH).

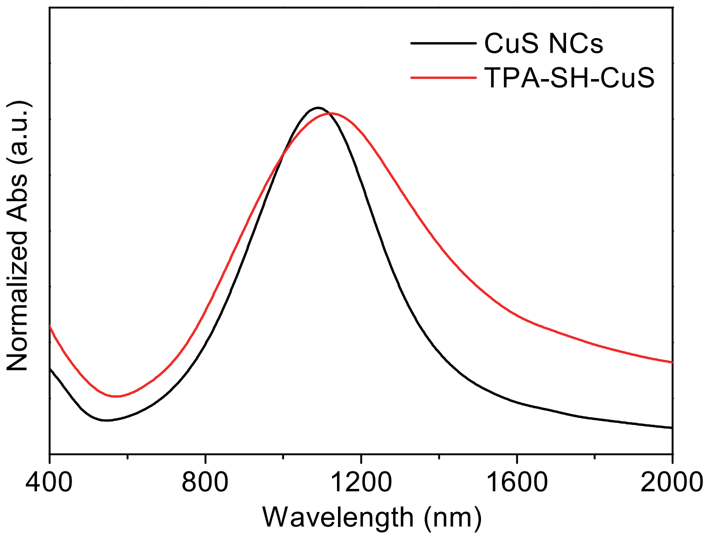

Supplementary Figure 10. Absorption spectra of CuS-TPA detrivative. Normalized absorption spectra of triphenylamine derivative (TPA-SH)-protected CuS NCs. TPA-SH-protected CuS NCs were synthesized to investigate the LSPR-induced hole transfer mechanism from the CuS phase to TPA, tracing the time-resolved formation of $TPA^{\bullet+}$ (Supplementary Figure 13).

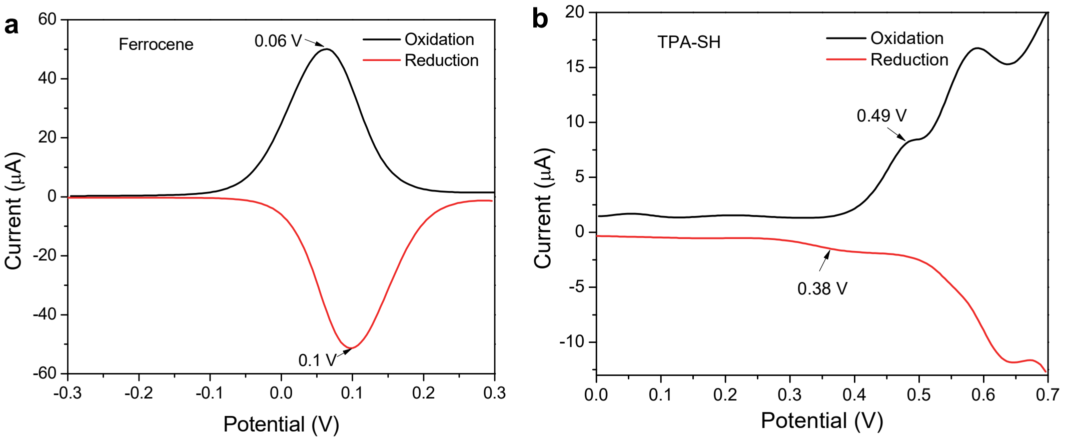

Supplementary Figure 11. Determination of HOMO level position of TPA-SH. Differential pulse voltammetry (DPV) measurements of **a,** ferrocene and **b**, TPA-SH. From the DPV measurements, the oxidation potential of TPA was determined to be 0.98 V *vs*. NHE. The hot hole transfer from the CuS phase to TPA was possible, as shown in Supplementary Figure 12.

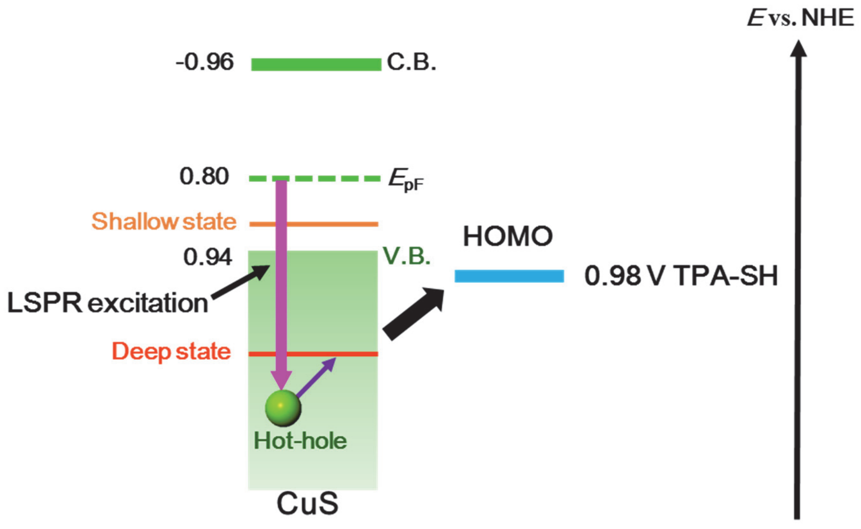

Supplementary Figure 12. Energy diagram of TPA-SH coordinated CuS. The relation of deep and shallow trapped state of CuS NCs and TPA-SH. HOMO: Highest occupied molecular orbital; CB: conduction band; VB: valence band; E_{pF}: Fermi level.

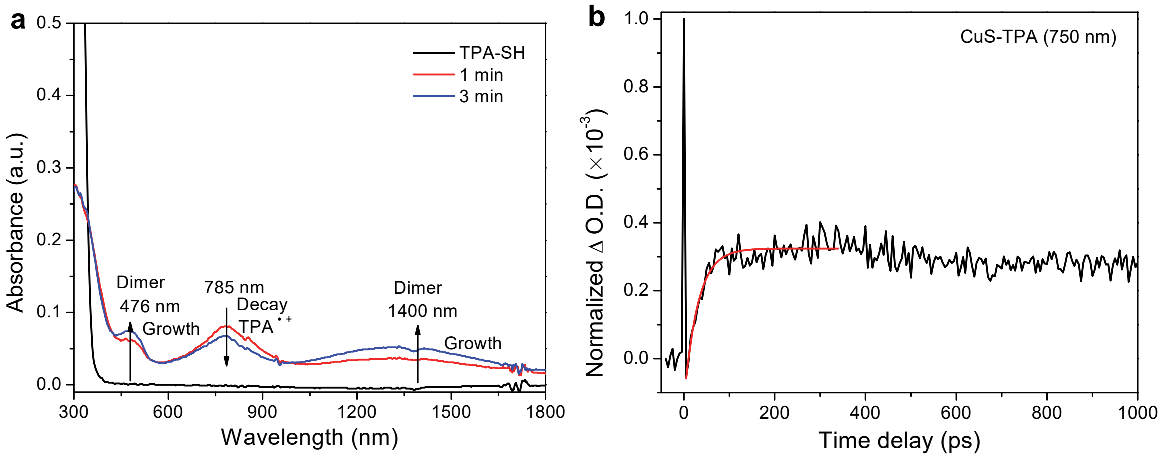

Supplementary Figure 13. Obsveration of TPA cation radical through plasmon-induced hole transfer for oxidating TPA. a, Absorption spectrum of TPA radical cations fabricated by adding oxidant (Cu^{2+}) to an acetonitrile solution of TPA. **b,** Kinetic profile of CuS-TPA at 750 nm upon excitation of CuS using a 1200-nm laser. The cation radical ($TPA^{\bullet+}$) rising component corresponds with stepwise hole transfer from CuS to TPA. Red line is the best fit.

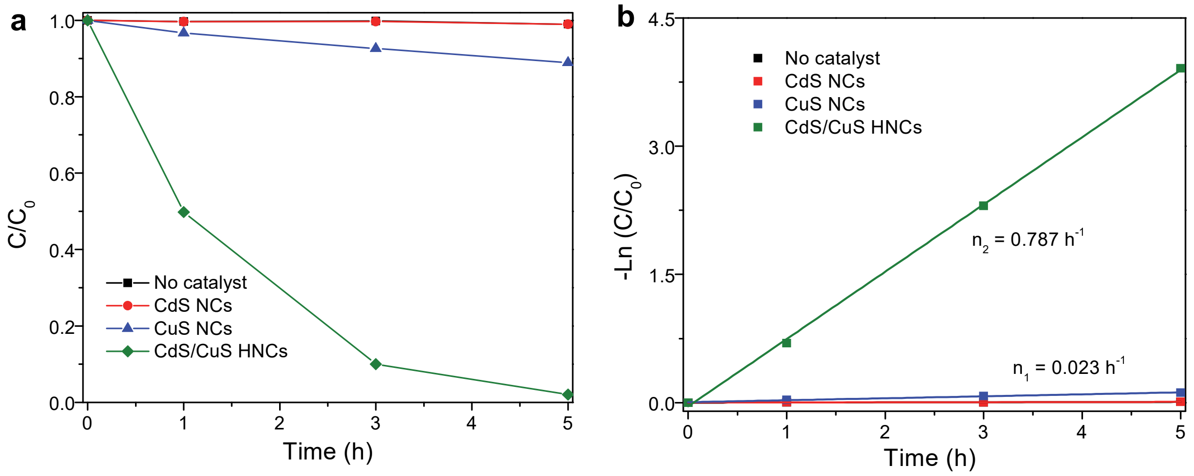

Supplementary Figure 14. The pollutant degradation at different NIR-irradiation time. Time-dependent change in **a**, MB concentration and **b**, estimation of MB degradation rates by reaction with CuS NCs, CdS/CuS HNCs, and CdS NCs under NIR light irradiation (Xe lamp; NIR light power density, 40 mW·cm^{-2}; wavelength, 750–1100 nm).

Supplementary Table 1. Lifetimes estimated from the kinetic traces of CuS NCs and CdS/CuS HNCs upon the excitaion of 1200-nm laser. Fitting procedures are described above.

Samples	Probe Wavelengths							
	1000 nm (ps)			560 nm (μs)		560 nm (ps)		
	τ_1 (ps)	τ_2 (ps)	τ_3 (ps)	τ_1 (μs)	τ_2 (μs)	τ_1 (ps)	τ_2 (ps)	τ_3 (ps)
CuS NCs	0.5	110	> 3000	1.7	-	90	489	-
CdS/CuS HNCs	0.4	80	> 3000	1.2	9.2	63	500	177

Supplementary References

1. Kanehara, M., Arakawa, H., Honda, T., Saruyama, M. & Teranishi, T. Large-scale synthesis of high-quality metal sulfide semiconductor quantum dots with tunable surface-plasmon resonance frequencies. *Chem. Eur. J.* **18,** 9230-9238 (2012).

2. Hu, K. *et al.*, Kinetic pathway for interfacial electron transfer from a semiconductor to a molecule. *Nat. Chem.* **8,** 853–859 (2016).

3. Pavlishchuk, V. V. & Addison, A. W. Conversion constants for redox potentials measured versus different reference electrodes in acetonitrile solutions at 25 °C. *Inorg. Chim. Acta* **298,** 97-102 (2000).
4. Lian, Z. *et al.*, Plasmonic silver quantum dots coupled with hierarchical TiO_2 nanotube arrays photoelectrodes for efficient visible-light photoelectrocatalytic hydrogen evolution. *Sci. Rep.* **5:** 10461 (2015).
5. Li, G., Lian, Z., Wang, W., Zhang, D. & Li, H. Nanotube-confinement induced size-controllable g-C_3N_4 quantum dots modified single-crystalline TiO_2 nanotube arrays for stable synergetic photoelectrocatalysis. *Nano Energy* **19,** 446-454 (2016).
6. Norris, D. J., Efros, A. L. & Erwin, S. C. Doped nanocrystals. *Science* **319,** 1776-1779 (2008).
7. Xu, Y. & Schoonen, M. A. A., The absolute energy positions of conduction and valence bands of selected semiconducting minerals. *Am. Mineral.* **85,** 543-556(2000).
8. Deng, X. *et al.* One-pot hydrothermal synthesis of CdS decorated CuS microflower-like structures for enhanced photocatalytic properties. *Sci. Rep.* **7**, 3877 (2017).
9. Nimmala, P. R., Yoon, B., Whetten, R. L., Landman, U. & Dass, A. $Au_{67}(SR)_{35}$ nanomolecules: characteristic size-specific optical, electrochemical, structural properties and first-principles theoretical analysis. *J. Phys. Chem. A* **117,** 504-517 (2013).
10. Sreenath, K., Suneesh, C. V., Ratheesh Kumar, V. K. & Gopidas, K. R. Cu(II)-mediated generation of triarylamine radical cations and their dimerization. an easy route to tetraarylbenzidines. *J. Org. Chem.* **73,** 3245-3251 (2008).
11. Quinn, J. J. & Ferrell, R. A. Electron self-energy approach to correlation in a degenerate electron gas. *Phys. Rev.* **112**, 812-826, (1958).
12. Echenique, P.M., Pitarke, J.M., Chulkov E.V., & Rubio, A. Theory of inelastic lifetimes of low-energy electrons in metals. *Chem. Phys.* **251**, 1–35, (2000).
13. Xie, Y. *et al.*, Metallic-like stoichiometric copper sulfide nanocrystals: phase- and shape-selective synthesis, near-infrared surface plasmon resonance properties, and their modeling. *ACS Nano* **7,** 7352-7369 (2013).
14. Ashcroft, N. W. & Mermin, N. D. *Solid State Physics Ch. 2* (Saunders Colledge, Philadelphia, 1976).
